# Neural representation of interval timing using electrocorticography

**DOI:** 10.1186/1471-2202-15-S1-P46

**Published:** 2014-07-21

**Authors:** Jonathan Flynn, Nitin Tandon, Harel Shouval

**Affiliations:** 1 Department of Neurobiology and Anatomy, UTHSC at Houston, TX 77030, USA; 2 Department of Neurosurgery, UTHSC at Houston, TX 77030, USA

## 

Interval timing refers to the cognitive ability to remember and process the length of time between two events, primarily on the scale of hundreds of milliseconds to tens of seconds [1]. It is critical for a wide range of behaviors, and deficits in interval timing are correlated with several disorders, including schizophrenia, ADHD, and autism [2]. While mechanisms for timing very short and very long periods (milliseconds and hours, respectively) have been extensively studied, the physiological basis for interval timing remains largely unknown [3]. It has often been assumed that specialized brain regions are used for estimating temporal intervals, but currently there is no significant evidence supporting this view. Recent experimental evidence in rodents indicates that the representation of timing might be distributed, and might even exist in primary sensory cortices [4]. For example, Shuler *et al*. (2006) found timing related neural spiking in V1, and subsequent work has found timing related activity even in cortical slices [5,6]. Our lab has proposed a model of how such temporal intervals can be represented and learned with recurrent neural networks [7]. These new experimental findings and related models suggest a theoretical framework in which interval timing has a distributed representation throughout the brain, and that this representation is formed via cortical plasticity.

Experimental work examining the mechanism of interval timing has been carried out in animal models, but not in humans. To explore the applicability of the distributed framework to humans, we have designed a set of experiments using electrocorticography (ECOG) in human patients. The patients were instructed to perform a basic interval-timing task, where they learned to respond to two different tones. For the lower frequency tone, patients were trained to press a button 1 second following the sound. For the higher frequency tone, patients were trained to press a button 3 second following the sound. Patients were given a ‘reward’ in the form of points based on how accurately they performed on the task. During the experiment, data was recorded from 50 to 128 subdural electrodes at a rate of 2000 Hz. The data was then filtered and band passed into frequency ranges that have been established to correlate with cognitive activity. Activity in different tone conditions was compared, primarily between the 1 and 3 second time points following the tone. Additionally, the activity immediately prior to reaction was compared to baseline. Preliminary power analysis has shown significant interval timing related activity in and around the auditory cortex. Activation in the anterior temporal lobe and the DLPFC also appear to correspond to timing behavior, suggesting that they may play some physiological role in interval timing. These combined findings support the basic assumptions of the distributed representation framework.
